# The Work Environment during Coronavirus Epidemics and Pandemics: A Systematic Review of Studies Using Quantitative, Qualitative, and Mixed-Methods Designs

**DOI:** 10.3390/ijerph19116783

**Published:** 2022-06-01

**Authors:** Anna Nyberg, Kristiina Rajaleid, Ingrid Demmelmaier

**Affiliations:** 1 Department of Public Health and Caring Sciences, Uppsala University, P.O. Box 564, SE-751 22 Uppsala, Sweden; ingrid.demmelmaier@pubcare.uu.se; 2 Stress Research Institute, Department of Psychology, Stockholm University, SE-106 91 Stockholm, Sweden; kristiina.rajaleid@su.se

**Keywords:** pandemic, epidemic, work environment, occupational health, mental health, PPE

## Abstract

We aimed to provide an overview of how work environment and occupational health are affected, and describe interventions designed to improve the work environment during epidemics and pandemics. The guidelines on Preferred Reporting Items for Systematic reviews and Meta-Analyses (PRISMA) were followed. The databases Cinahl, Medline, PsycInfo, and Web of Science were searched for population: working population; exposure: coronavirus epidemic or pandemic; and outcome: work environment, in articles published until October 2020. Quality assessment was based on a modified version of the Mixed Methods Appraisal Tool (MMAT). After deduplication 3711 articles remained, of which 530 were selected for full-text screening and 119 for quality assessment. After the exclusion of studies that were low quality, 95 remained, of which 85 focused on healthcare personnel and 10 on employees in other industries; 73 used quantitative methods and 22 used qualitative or mixed methods; the majority were based on cross-sectional data. Healthcare staff experienced increased job demands, poor leadership, and lack of resources (personal protective equipment, personnel, and competence). High demands and work with infected patients were associated with negative mental health outcomes. There was a lack of studies assessing interventions, studies from industries other than healthcare, and studies of high quality.

## 1. Introduction

The outbreak of SARS-CoV-2 virus in China in late 2019 rapidly developed into an epidemic, and in March 2020, the World Health Organization declared a state of global pandemic [1]. The spread of disease and restrictions to counteract the spread affected all of society, including working life, educational systems, and healthcare organizations. COVID-19 developed to be a major occupational health risk for employees not only in healthcare [2] but also in the service, manufacturing, and agriculture industries [3,4]. Previous coronavirus epidemics, such as severe acute respiratory syndrome (SARS) with an outbreak in 2003 and Middle East respiratory syndrome (MERS) with an outbreak in 2012, had a similar impact on working life and health, although not as widespread throughout societies worldwide. The COVID-19 pandemic is likely to be followed by new epidemics and pandemics affecting working life and employees in the decades to come, and employers and society need to be prepared to handle new health crises. The establishment of occupational safety policies to counteract the negative health effects of new epidemics and pandemics need to be based on scientific evidence. Therefore, there is a need to systematically review the current research on how corona epidemics and pandemics impacted working life and employee health, and what measures aimed at counteracting negative health effects on employees were effective. Several reviews of the work environment during the COVID-19 pandemic have been published [5,6,7,8,9,10,11,12]. However, most reviews focused solely on the impact on healthcare organizations [6,7,8,10,11,12], and it is important to include studies describing other labor market industries and how they are affected by epidemics and pandemics. Two reviews had a broader scope: an umbrella review from Canada [9] and a systematic review from Brazil [5]. However, the umbrella review did not evaluate the methodological quality of the included studies, and the systematic review did not include intervention studies targeting work environment factors. Moreover, previous research reviews only included studies of the work environment during COVID-19, possibly missing important knowledge drawn from previous coronavirus epidemics. With the present systematic review of studies, focusing on how the work environment and employee health are affected during an epidemic or pandemic caused by coronavirus (COVID-19, SARS, and MERS), and the effect of interventions to improve work environment and employee health, we intend to fill this knowledge gap. This work is based on a systematic review commissioned by the Swedish Agency for Work Environment Expertise.

### Aim

The aim of the present study was to systematically review research on work environments and employee health during coronavirus epidemics or pandemics. The specific research questions were: During an epidemic or pandemic caused by a coronavirus,

1. How is the work environment affected?
2. What are the associations between work environment factors and employee health?
3. What are the effects of interventions to improve the work environment or health?

## 2. Methods

We followed the guidelines on Preferred Reporting Items for Systematic reviews and Meta-Analyses (PRISMA) [13]. A professional at the University Library at Uppsala University, Sweden, conducted the literature search in the start of October 2020 and a protocol was registered in Prospero on the 29th March 2021 (no CRD42021229165). A principal investigator (AN) and two additional researchers (ID, KR) made up the core review team and two research assistants (YL, WL) and an expert on qualitative research (UW) provided input in particular parts of the review process.

### 2.1. Search Strategy and Study Selection

The search strategy was developed by the core review team in collaboration with a librarian at Uppsala University. The databases Cinahl, Medline, PsycInfo, and Web of Science were searched for articles published up until October 2020. The search strategy was based on a combination of terms identifying exposure (healthcare epidemic or pandemic), population (working population), and outcome (work environment). The full search strategy is given in Appendix A.

The inclusion criteria for the present study were:

(1) Language: Swedish or English;
(2) Population: The working population;
(3) Exposure: Corona virus epidemic or pandemic; and
(4) Outcome: Work environment. Only original articles published in peer-review journals were considered.

First, the titles and abstracts identified by the search were screened against our inclusion criteria by two researchers or research assistants in the review team. Discrepancies or uncertainties were discussed and resolved in the core review team. In case the titles and abstracts did not provide enough information, the articles were moved forward to the next step of the selection process. Next, full-text articles were screened by two members of the core review team according to the same procedure as for the previous step. Reasons for exclusion of an article were noted. We used the software tool Covidence (www.covidence.org, accessed on 29 September 2020) for the selection of abstracts and full-text articles. Next, the data of the selected articles were extracted to Excel sheets.

### 2.2. Quality Assessment

The data extracted from the full-text articles to the Excel sheets were author, journal, year of publication, country of origin, population, response rate, research question, study design, exposure, outcome, confounders considered, follow-up time, analytical strategy, and main results. The quality assessment was based on a slightly modified version of the Mixed Methods Appraisal Tool (MMAT) [14]. Different assessment criteria were considered for the different study designs. One researcher was responsible for the quality assessment of each study, but calibration between assessors and studies was carried out continuously throughout the assessment process. When uncertainties arose, assessment was conducted by discussion between the three members of the core review team. Each study was given a score between one and four or between one and five, depending on the study design. Based on this score, the study was further assessed to be of low, medium, or high quality. See Appendix A for a full description of the quality assessment process. Only studies of medium or high quality were considered in the summary of results, discussion, and conclusion, but low-quality studies are also listed in the tables.

### 2.3. Summary of Study Results

The majority of studies were based either on cross-sectional or qualitative data. Given the quality of the evidence and the diversity of studies included in the present systematic literature review, a meta-analysis was not feasible. The results are instead carefully presented in seven exhaustive tables. Work environment factors that were reported in several quantitative and qualitative studies are described and highlighted in the discussion section.

## 3. Results

**Figure 1:**
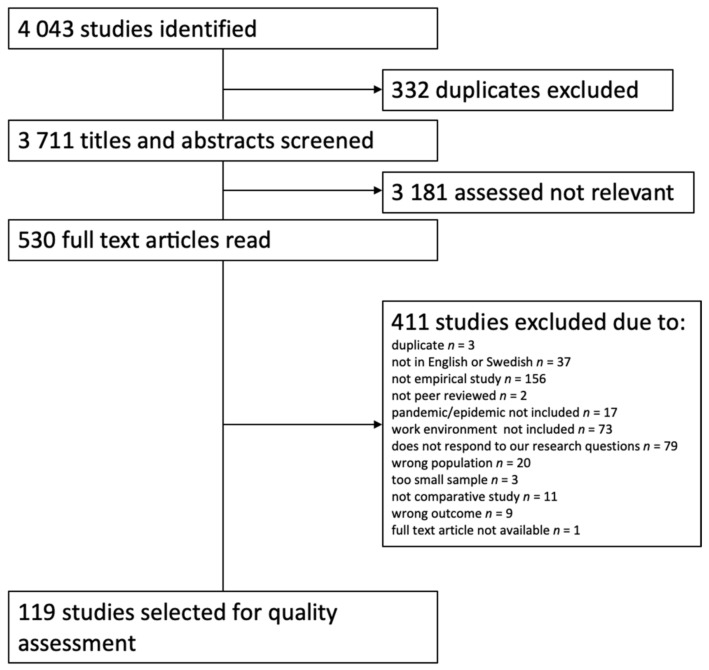
Flowchart of the selection and exclusion of articles.

In the literature search 4043 hits were recorded, of which 3711 remained after deduplication. The titles and abstracts of these articles were screened for eligibility and 530 were moved forward for full-text screening. During this step of the process, an additional 411 articles were excluded (primarily because of not being an empirical study) and 119 were passed on to the stage of quality assessment (see Figure 1).

Of the 119 selected articles, 24 were excluded due to low quality [15,16,17,18,19,20,21,22,23,24,25,26,27,28,29,30,31,32,33,34,35,36,37,38]. Of the remaining 95 studies, 85 investigated the healthcare industry and 10 studies focused on other industries. The results are presented separately for the healthcare industry and other industries below.

### 3.1. The Healthcare Industry

**Figure 2:**
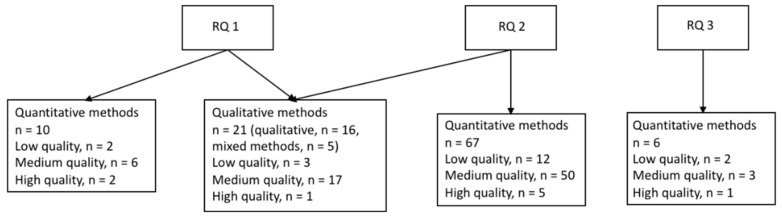
Studies of the healthcare industry sorted by research question and methodology. Research questions (RQs): During an epidemic or pandemic caused by a coronavirus: (1) How is the work environment affected? (2) What are the associations between work environment factors and employee health? (3) What are the effects of interventions to improve the work environment or health?

Of the 85 studies focusing on healthcare workers (HCWs, reported in Table 1, Table 2, Table 3 and Table 4), 67 studies were based on quantitative data (Table 1, Table 2 and Table 3) and 18 on qualitative or mixed-methods designs (Table 4). The quantitative studies are presented separately for research question 1, 2, and 3 below followed by the results from the qualitative and mixed-methods studies, which were combined for research question 1 and 2 as they were often assessed simultaneously and were not possible to separate in the synthesis of the results (see Figure 2). We did not identify any qualitative or mixed-methods studies assessing research question 3.

#### 3.1.1. Results from Quantitative Studies

RQ 1: How is the work environment affected by an epidemic or pandemic?

There were eight studies of medium or high quality that used quantitative methodology to investigate how the work environment was affected by a pandemic or epidemic [39,40,41,42,43,44,45,46] (see Table 1). One of them included data from four continents: four studies were from Europe, one from North America, one from Australia, and one from Asia. Six focused on the work environment during COVID-19 and two during SARS.

In several studies, workload was reported to be increased during the pandemic [39,41,42,44], particularly in high-risk sectors [39], by those working close to infected patients [41], by nurses [42], and by women [39]. In contrast, primary healthcare nurses in Australia reported decreased work hours and threats of termination since the onset of the COVID-19 pandemic [40]. One study from the UK reported that telemedicine was efficient and without an associated increased workload [43]. Differences in personnel shortage problems, provision of personal protective equipment (PPE) and PPE training, and staff diagnosed with COVID-19 were reported between Asia, Europe, North America, and South America [45]. Nurses reported more negative factors in the work environment, such as poor information, insufficient infection control measures, and lack of appreciation by employer, than doctors [42,46].

RQ2: Associations between work environment and health during an epidemic or pandemic.

Of the 55 quantitative studies of medium to high quality that investigated associations with health outcomes, 44 investigated associations with mental ill-health [47,48,49,50,51,52,53,54,55,56,57,58,59,60,61,62,63,64,65,66,67,68,69,70,71,72,73,74,75,76,77,78,79,80,81,82,83,84,85,86,87,88,89,90], 4 associations with physical complaints due to PPE [91,92,93,94], and 7 associations with risk of healthcare infection [95,96,97,98,99,100,101] (see Table 2). One of the studies was global, 12 were from Europe, 12 from North America, 26 from Asia, 1 from South America, and 3 from the Middle East. Most studies, 36 of them, investigated the work environment and health during COVID-19, 15 during SARS, and 4 during MERS. Three studies compared mental ill-health between healthcare workers and workers in other professions and found that healthcare workers reported increased mental ill-health in comparison [47,53,88]. A large number of the studies focusing on mental health outcomes reported associations between working in the frontline (i.e., working with patients infected or suspected to be infected) and symptoms of depression, anxiety, emotional exhaustion, burnout, sleep disturbances, or post-traumatic stress syndrome [48,50,54,55,57,63,65,67,70,75,81,82,84,86,89,90]. Among these employees, several work stressors were found to be associated with mental health outcomes. For example, longer working hours or overtime work [50,53,68,77,87,88] and type of shift/higher frequency of night shifts [60,64,87] were associated with increased symptoms of mental ill-health. Other factors associated with mental health symptoms were not having adequate knowledge and training to meet the work demands [50,62,67,70,71,83], lack of PPE [61,65,70,77,80,85], fear of being infected by healthcare [61,83], and experiencing stigmatization for working with infected patients [59,69,74]. Lastly, lack of organizational resources, such as manpower [83] or hospital resources for the treatment of infected patients [60] and lack of organizational and social support at work [50,52,62,65,67,84], was associated with negative mental health outcomes.

Seven studies focused on the risk of healthcare infection in healthcare personnel in relation to exposure to infected patients [95,96,97,98,99,100,101]. Higher risk of infection was found to be associated with working within a meter of an exposed patient [99], intubating [96,97], or performing chest compression [101] on a patient, having contact with respiratory secretion of an infected patient [100,101], inadequate training in infection control [95,101], and not using adequate PPE [95,96,100,101].

Four studies of medium or high quality [91,92,93,94] investigated the association between use of PPE and skin injuries or headaches. In three studies [91,92,93], an association between use of PPE and skin injuries was reported and in one study [94], an association with headaches was found.

RQ3: Interventions to improve the work environment during an epidemic or pandemic.

Four studies that measured the effects of interventions to improve the work environment and health were assessed to be of medium or high quality [102,103,104,105]. Three of these [103,104,105] focused on infection control, of which two investigated the effects of infection control training and one compared infection rates between hospitals with and without certain infection control measures. All interventions showed effects on infection. The fourth study [102] evaluated the mental health effects of an intervention targeting several organizational factors such as training, resources, infection control, and support teams. The intervention was found to protect the mental health of healthcare staff.

#### 3.1.2. Results from Qualitative Studies

RQ1 and 2: The work environment during an epidemic or pandemic and its association with employee health.

There were 18 studies of medium to high quality that explored healthcare staffs’ experiences using a qualitative or mixed-methods designs [106,107,108,109,110,111,112,113,114,115,116,117,118,119,120,121,122,123]. Two studies were performed in Europe, nine in Asia, five in North America, and two in the Middle East. Twelve of the studies were performed during the COVID-19 pandemic, four during SARS, and two during MERS (see Table 4). All studies included a thematic analysis of qualitative data based on interviews or open-ended questionnaire items. The mixed-methods studies also included some quantitative elements.

Healthcare staff described high quantitative demands in terms of increased workload, intense work, large patient volumes, increased work shifts, and more administration [106,107,110,111,113,114,122] causing distress or burnout. In a mixed-methods study, self-reported burnout symptoms were found in 10–18% of 468 emergency physicians during the first 10 weeks of the COVID-19 pandemic [107]. Staff also described high qualitative demands, expressed as difficult tasks [108,111,123], new routines [107], collaborating with inexperienced staff [123], and working in a totally new context [113]. They struggled to stay updated on new, constantly changing guidelines [110] and to manage uncertainty [112,115]. Additional themes related to working with infected patients were feeling powerless [113], anxiety and suffering in staff working with seriously ill and dying patients [111,122], and decreased quality of care [108,116]. In several studies, healthcare staff reported a lack of PPE and a fear of becoming infected [106,107,108,110,111,112,114,115,121]. Among staff using PPE, there were reports about exhaustion and discomfort caused by the equipment [113,120,122]. Social stigma and being avoided by family and others due to patient contact was also described [108,117,120,121]. Several studies included staff’s descriptions of deficits in work organization: a chaotic workplace [110], insufficient preparedness for the pandemic [121], lack of information provided to staff [109,118,119], mismatch of nursing competencies and tasks [109], and an unfair work distribution [108]. Other studies included descriptions of healthcare staff’s suggestions for future improvements: involving all actors in developing communication strategies and being open about risk communication [118]. Furthermore, the development of effective work routines [108,109,122], increased attention given to staff’s mental and physical health [109], and disaster rescue training were suggested [114]. Among psychotherapists working remotely, this was perceived as challenging by 80%; nonetheless, 65% considered this to be the future core business for them [116].

Some positive aspects were described: healthcare staff experienced satisfaction and professional development during the SARS epidemic [106] and the COVID-19 pandemic [113,114,122]. Increased flexibility and less family–work conflict, thanks to remote work, were described by psychotherapists [116]. Social support [113,122] and working towards a common goal [106] were described as positive.

### 3.2. Industries Other Than Healthcare

**Figure 3:**
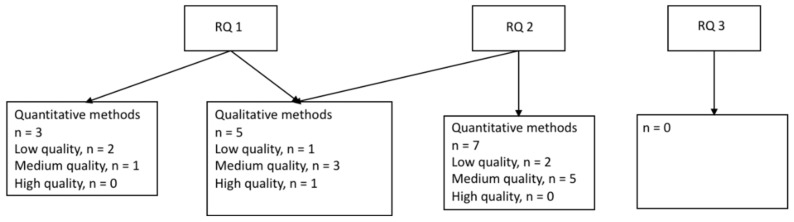
Studies of industries other than healthcare, sorted by research question and methodology. Research questions (RQs): During an epidemic or pandemic caused by a coronavirus: (1) How is the work environment affected? (2) What are the associations between work environment factors and employee health? (3) What are the effects of interventions to improve the work environment or health?

Of the ten studies focusing on industries other than the healthcare industry (reported in Table 5, Table 6 and Table 7), six studies were based on quantitative data (Table 5 and Table 6) and four on qualitative or mixed-methods designs (Table 7). The quantitative studies are presented separately for research question 1 and 2 below, followed by the results from the qualitative and mixed-methods studies presented together for the same research questions (see Figure 3). No studies of interventions aiming to improve the work environment or employee health in industries outside healthcare were identified.

#### 3.2.1. Results from Quantitative Studies

RQ1: How is the work environment affected by a pandemic or epidemic?

Only one study of high enough quality matched the inclusion criteria [124]. The study was from Brazil and investigated changes in working conditions among a variety of professions, such as teachers, social workers, psychologists, physicians, and lawyers, working with child health and security. The results showed that their working hours had decreased during the COVID-19 pandemic compared to before.

RQ2: Associations between the work environment and health during an epidemic or pandemic

Five studies, all with a cross-sectional research design, performed in Asia, and assessed to be of medium quality, met the inclusion criteria [125,126,127,128,129]. The samples and research questions were heterogenous. In male police officers in Pakistan during the COVID-19 pandemic, an association between work–family conflict and work-related stress was found [125]. In a Chinese study, no association between where the employees worked (at home, at the office, or both) and mental ill-health was found, with the exception that those who alternated between the office and home reported less somatization symptoms [127]. Another Chinese study reported an association between improved hygiene routines during COVID-19 and lower levels of self-reported stress among employees [128]. A Japanese study found a positive association between the number of preventive infection measures implemented at the workplace, and the level of fear and anxiety related to COVID-19, a result that the authors stated may be due to a higher awareness about the disease at workplaces with a larger number of preventive measures [126]. Last, a study from Hong Kong found associations between dissatisfaction with infection control measures, perceived infection risk, and lower health-related quality of life [129].

#### 3.2.2. Results from Qualitative Studies

RQ1 and 2: The work environment during an epidemic or pandemic and its association with employee health

Four studies of medium or high quality explored experiences among staff in industries other than healthcare using a qualitative or mixed-methods design: three in the educational system [130,131,132] and one in social work [133]. Two studies were performed in North America (SARS and COVID-19, respectively), one in Europe (COVID-19), and one in Australia (COVID-19) (see Table 7). The teachers described how the quick transition to remote teaching during the COVID-19 pandemic increased their workload [132] and introduced uncertainty [130] and techno stress [131]. Furthermore, they reported how they lost the opportunity to teach hands-on skills [131,132] and worried about students’ learning outcomes [130,132] and wellbeing [130]. The transition to remote teaching was perceived as “demanding” or “very demanding” by 49% of the educators at a physician assistant program in North America [131]. However, they also described how they adjusted and found positive aspects of remote teaching such as using new pedagogies and synchronous teaching across remote sites [132] and increased flexibility, sometimes at the cost of difficulties in home–work balance, during the workday [130]. Social workers at a hospital described how the SARS epidemic influenced their professional roles by increasing the emotional awareness of patients’ families and other professionals and problems regarding information about guidelines and their implementation [133].

## 4. Discussion

To the best of our knowledge, this is the first systematic review of studies describing the impact of healthcare epidemics and pandemics (COVID-19, MERS, and SARS) on the work environment and employee health, and the effect of interventions, including industries within and outside healthcare organizations. Previous reviews have reported healthcare to be the most thoroughly studied industry [5,9] and the present review confirms this is still the case.

Based on the findings in 95 original studies (85 within healthcare and 10 in other industries), we found unambiguous evidence for a negative impact on healthcare staff in terms of increased demands such as excessive workload and difficult tasks. Healthcare staff reported that they needed to adapt quickly to new routines and collaborations, managing insecurity and a lack of resources. There was a need for strong communicative leadership and provision of social support. Mental ill-health among healthcare staff was associated with exposure to infected patients, high demands, lack of PPE, lack of competence, lack of social support at work, feeling stigmatized, and perceiving a high risk of becoming infected. Nurses reported worse mental health compared to other healthcare professions. A few intervention studies, all within healthcare organizations, were identified. They evaluated training and new routines to reduce the risk of disease transmission and reported positive results. The small number of studies performed in industries outside healthcare found mental ill-health to be associated with insufficient preventive measures at the workplace, high workload, work–family conflict, and not being able to work remotely. A few studies exploring teachers’ experiences reported an increased workload due to the quick transition to remote teaching but also positive aspects of learning new teaching methods that they wished to integrate in their regular teaching after the pandemic.

Several original articles focusing on the COVID-19 pandemic, published after the search in October, 2020, support the findings in the present review: increased working hours and occupational stigma were associated with worse mental health and intention to leave among Taiwanese nurses [134]; mental health among primary healthcare workers in China was negatively affected by pandemic-related work stress; however, such stress was attenuated by social support and resilience [135]; and, finally, a Swedish longitudinal study found negative changes in healthcare staff’s working conditions and their possibility to recover after comparing their ratings before and after the first wave of COVID-19 [136].

### 4.1. Overall Strength of the Evidence

The large number of articles published since COVID-19 started to spread demonstrate the immediate response in the research community to the pandemic. Evidence started to accumulate within a few months from the outbreak, and a picture is emerging of how the work environment and employees’ health have been affected so far. The evidence from the healthcare sector shows a coherent picture from quantitative and qualitative data from studies worldwide of a strong impact on this industry.

### 4.2. Limitations of the Current Evidence

The weak study designs and overall low methodological quality of the included studies indicate that quantity was prioritized ahead of quality during the first few months of the COVID-19 pandemic but also during previous healthcare epidemics. The speed of the review and publication process, indicated by the dates of the reception, acceptance, and publication of articles, suggests that the peer-review process may have been less stringent than what is normally the case. Most of the studies that met the inclusion criteria for the present systematic review were based on convenience samples and cross-sectional designs. Furthermore, most studies used non-validated measures of exposure and also of the outcomes, and potential confounders were often not considered in the analyses. These methodological flaws prevent any conclusions about the causality between the work environment factors and employee health. The SARS and MERS epidemics were included in the present review because, with time having passed, we expected that studies with longitudinal designs and of higher quality had been published, providing an opportunity to learn from previous healthcare epidemics. However, few longitudinal follow-up studies of the previous healthcare epidemics were identified in our literature search, demonstrating a missed opportunity to learn about the long-term consequences of such epidemics.

### 4.3. Overall Gaps in Knowledge and Research

Longitudinal studies are needed to follow changes over time, accumulating knowledge that may enable conclusions about the causality and effects in different groups. Furthermore, it is important to develop and evaluate interventions using experimental designs to establish effective measures to enhance the work environment and health as new epidemics and pandemics evolve. In healthcare, employees report experiencing stigma for working with infected patients. More research is needed on this topic, and how to protect healthcare workers from such an extra burden during a health crisis. There is a paucity of studies from industries other than healthcare investigating the work environment and employee health during and after epidemics and pandemics. There is, for example, a need to study the long-term effect of remote work to evaluate both positive and negative impacts. There has, in general, been a larger focus on risks and stressors in the work environment and less research on possible positive changes to the work environment that can inform the development of the new working life past COVID-19.

### 4.4. Strengths and Limitations of the Current Review

The present review was conducted in accordance with the PRISMA guidelines for developing and reporting systematic research reviews. The search strategy was developed in a collaboration between researchers and a search specialist at Uppsala University library. A large number of studies about the work environment and health during the outbreaks of COVID-19, SARS, and MERS had been published by the search date in October 2020. The authors of the present review screened, together with two research assistants, the 4043 hits and each study was independently evaluated by at least two people in the team. Many full-text articles were read and discussed in the core assessment team. An updated search was conducted at the end of January 2021, generating an additional 2915 hits, which was too large a number of extra articles to be handled with sustained methodological stringency within the time frame of the commission by the Swedish Agency for Work Environment Expertise. A quick assessment of the new hits gave us the primary impression that most of the literature that had been published between October 2020 and January 2021 suffered from similar methodological limitations to the studies published until October 2020 and included in this review. Nevertheless, it cannot be ruled out that we could have identified additional relevant studies of higher quality had we had the possibility of prolonging the time frame for our literature search.

The search strategy was developed carefully, with consideration of capturing relevant studies while simultaneously obtaining an amount of hits in the search result that was feasible to handle within the given time frame. We used MESH terms and search terms for work environment but not for specific work environment risks. We tried to develop such search terms, but because it was impossible to draw the line between which ones to include and which ones to leave out, we decided to only use broader terms. Although this was the most feasible solution, it means that if a study did not include search terms for work environment while still investigating an aspect of this, it was not included in the present review (see the search strategy in Appendix A).

The inclusion of different study designs in the present review provided a nuanced picture of current research but made the evidence more difficult to synthesize. The results across studies with different designs, however, were overall coherent and pointed in the same direction. Using the MMAT meant that an assessment of studies with different designs was parsimonious but possibly not thorough enough to capture all important aspects of methodological quality. It did not, for example, include an assessment of causality. While this assessment tool matched the quality of the evidence of the research field at the time the search was conducted, it also means that the bar for a study to qualify as medium or high quality was quite low.

### 4.5. Implications for Future Research

Based on the identified knowledge gaps, longitudinal studies are needed to assess the work environment and employee health over time during and after epidemics or pandemics. It is evident that the work environment and employee mental health were affected among healthcare personnel during the acute phases of the pandemic, particularly those working in the frontline, but research on the long-term effects on health after the pandemic is over appears crucial. Preferably, studies could make use of data on work environment factors and health collected before epidemics and follow the samples during and after pandemics, building up longitudinal data to study changes in these measures over time. Intervention studies aimed at mitigating the negative effects on the work environment and employee health are important to inform policy development in case of future epidemics and pandemics. To increase the quality and comparability of studies of the work environment during pandemics and epidemics, validated measures of work exposure and health outcomes need to be used and confounders carefully accounted for in statistical analyses. Finally, more research focusing on industries other than healthcare is needed. Examples are the educational industry, also widely affected by the pandemic, and the service and culture sectors (that were affected economically) and many white-collar industries in which workers quickly transitioned from office to remote work.

## 5. Conclusions

During the first months of the COVID-19 pandemic, a large number of cross-sectional studies, quantitative and qualitative, on the work environment and health, mainly within the healthcare industry, were published. The weak study designs and overall low methodological quality limited the opportunity to learn about the long-term health effects of changes in the work environment during the pandemic. However, there was a remarkable accordance in the results of the situation in the healthcare industry from studies spanning several continents and using different methodologies, painting a picture of increased quantitative and qualitative demands, lack of personal and organizational resources to meet these demands, fear among personnel of being infected, and experienced stigma related to work with infected patients.

### 5.1. Practical Implications

The possible long-term health effects of the increased demands and limited resources that have characterized the work environment for healthcare employees during acute phases of the COVID-19 pandemic need to be followed up clinically and in research.

### 5.2. Key Messages

- The work environment and mental health were strongly affected in healthcare employees during the first months of the COVID-19 pandemic.
- Longitudinal studies are needed to evaluate the possible long-term consequences of severe and long-lasting stressors in the work environment during the COVID-19 pandemic within healthcare.
- Studies evaluating the effects of interventions aiming to improve the work environment and mitigate negative health effects are needed to prepare for future pandemics.
- Studies of higher quality with regards to sampling strategies, exposure and outcome measurements, control of possible confounders, etc. are needed to move the research field forward.
- Studies from industries outside healthcare are needed to obtain a broader picture of how pandemics affect the work environment of entire labor markets.

## Figures and Tables

**Table 1:** Overview of quantitative studies assessing how the work environment is affected by an epidemic or pandemic (COVID-19, SARS, or MERS) in the healthcare industry (research question 1).

No	Author (Year)	Country	Population	Design, N (% Women)	Exposure (Pandemic)	Result	Subgroup Comparison	Overall Quality
1	Felice (2020)	Italy	HCWs, mainly physicians, in northern Italy	Cross-sectional survey, n = 388 (61%)	COVID-19	Females and respondents working in high-risk sectors were more likely to rate psychological support as useful and workload as increased.	Gender, occupation, standard vs. high-risk sector	Medium
2	Halcomb (2020)	Australia	Primary healthcare nurses	Cross-sectional survey, n = 637 (96%)	COVID-19	Nearly half of the respondents reported either decreased hours of employment, threatened termination, or actual termination of employment since the onset of the pandemic. Most respondents reported that they had sufficient knowledge about COVID-19 but that they never or only sometimes had access to sufficient PPE.	None	High
3	Koh (2005)	Singapore	HCWs	Cross-sectional survey, n = 10,511 (82%)	SARS	More than half reported an increased workload. Non-SARS-affected hospitals had a higher increase in workload than SARS-affected hospitals. Being exposed to SARS daily was associated with a higher increase in workload than being exposed less often. Nurses and several other occupational groups reported a higher increase in workload than doctors.	Occupational groups, hospitals	High
4	Kramer (2021)	Germany	HCWs	Cross-sectional survey, n = 3669 (61%)	COVID-19	More nurses reported a high increase in workload, not being sufficiently informed about the pandemic, feeling left alone by the employer, and that the employer had not taken appropriate measures, informed appropriately, or were prepared for the pandemic compared with doctors and other occupational groups. More nurses than doctors and others further felt little appreciation from the management, were afraid of catching the virus, and more often reported that they would not continue working in the healthcare industry after the COVID-19 pandemic. There were several statistically significant differences in work environment factors in comparisons between ICU, ER, COVID-19 wards, and other wards.	Doctors, nurses, others ICU, ER, COVID-19 ward compared with others	Medium
5	Sarma (2020)	India	HCWs	Cross-sectional survey, n = 110 (40%)	COVID-19	In total, 84.5% of the participants were concerned about the risk of infection to self and family and 56.4% were disturbed by the lack of any concrete protocol for patient management. Less staff availability, delay in discharging duties toward their patients, and increased workload were other concerns.	None	Low
6	Semaan (2020)	Global	HCWs (maternal and newborn health professionals)	Cross-sectional survey, n = 714 (not reported)	COVID-19	The percentage of respondents who reported available/updated guidelines, access to COVID-19 testing, and dedicated isolation rooms for confirmed/suspected COVID-19 maternity patients was higher in high-income countries than in low- and middle-income countries (difference not tested statistically).	High- compared with low- and middle-income countries	Low
7	Smrke (2020)	UK	HCWs (physicians, and nurses in rare cancer care)	Cross-sectional survey, n = 18 (not reported)	COVID-19	In total, 75% of the planned face-to-face appointments in rare cancer care were converted to telemedicine. Clinicians found telemedicine efficient, with no associated increased workload.	None	Medium
8	Spiller (2020)	Switzerland	Nurses and physicians	Two independent cross-sectional samples: during the COVID-19 outbreak and after its flattening n = 812 (71%)	COVID-19	Nurses and physicians reported, in both samples, that they worked more during the pandemic than before. They also suffered more from anxiety and burnout.	None	Medium
9	Teoh (2020)	Asia, Europe, North America, and South America	HCWs (urology staff)	Cross-sectional survey, n = 1004 (18%)	COVID-19	A higher number of staff had been diagnosed with COVID-19 in Europe and North America than in the other countries; European respondents cited the highest percentage of personnel shortage problems followed by South America and Asia. Provision of PPE and PPE training also differed by continent.	Africa, Asia, Australia/NZ, Europe, North America, South America	Medium
10	(2005)	Canada	HCWs	Cross-sectional survey, n = 300 (74%)	SARS	Nurses relied more on peer support than doctors, felt less informed and less involved in decision-making than doctors felt, and were more likely to report that infection control procedures were not strict enough.	Doctors/Nurses	Medium

HCWs: Healthcare workers; PPE: Personal protective equipment.

**Table 2:** Overview of quantitative studies assessing the associations between work environment factors and health (research question 2) in the healthcare industry during an epidemic or pandemic (COVID-19, SARS, or MERS).

No	Author (Year)	Country/ Pandemic	Population	Design, N (% Women)	Exposure	Outcome	The Association between WE and Health	Subgroup Comparison	Overall Quality
1	Alraddadi (2016)	Saudi Arabia/ MERS-Cov	HCWs	Retrospective cohort study, n = 292 (64%)	Working in units that treated MERS-CoV patients	MERS-CoV antibodies	Attack rate in the medical intensive care unit of 11.7%, emergency department of 4.1%, neurology unit (with no known MERS-CoV patients) of 0%. Those who had undergone infection control training specific to MERS-CoV had a lower risk of infection. Always covering the nose and mouth with a medical mask or N95 respirator when caring for MERS-CoV patients was associated with a lower risk of infection.	Occupation: radiology technicians attack rate 29.4%, nurses 9.4%, respiratory therapists 3.2%, physicians 2.4%; clerical staff 0%, patient transporters 0%. Gender: no difference by sex	Medium
2	Bai (2004)	Taiwan/ SARS	HCWs and administrative personnel	Cross-sectional survey, n = 338 (51%)	Administrative vs. healthcare personnel, quarantined vs. not	Several mental health outcomes	Association between being quarantined and acute stress disorder. In contrast to administrative personnel, healthcare workers reported experiencing significantly more insomnia, exhaustion, and uncertainty about the frequent modifications of infection control procedures.	Administrative versus healthcare personnel, quarantined versus not quarantined	Medium
3	Buselli (2020)	Italy/ COVID-19	HCWs in a major university hospital in Italy	Cross-sectional survey, n = 265 (69%)	ICU staff/frontline staff	Symptoms of anxiety and depression	Association between working in the frontline and reported symptoms of anxiety but not symptoms of depression.	ICU staff/frontline staff	Medium
4	Chatterjee (2020)	India/ COVID-19	HCWs	Case-control study, n = 378 cases (42%) and n = 373 controls (49%)	Use of PPE, performing endotracheal intubation	qRT-PCR test result	Increased risk if never used PPE (OR = 5.33 with 95% CI 2.27–12.48)) and if performing endotracheal intubation (OR = 4.33, 1.16–16.07).	None	Medium
5	DeSio (2020)	Italy/ COVID-19	Physicians in Rome and Florence	Cross-sectional survey, n = 695 (45%)	Caring for COVID-19 patients versus not	Psychological distress (GHQ-12) and perceived well-being (WHO-5)	Higher odds of symptoms of mental ill-health among physicians working in areas most affected by COVID-19 compared with physicians working in areas less affected by COVID-19.	Areas more or less affected by COVID-19	Medium
6	Elbay (2020)	Turkey/ COVID-19	Physicians	Cross-sectional survey, n = 442 (57%)	Frontline work, workload, competence, support	Depression and anxiety (DASS-21)	Association between working in the frontline and sum score of depression and anxiety; among frontline workers, the association between higher weekly working hours, higher number of COVID-19 patients cared for, lower level of support, and lower level of experienced competence on the one hand and sum score of depression and anxiety on the other.	Working in frontline versus not	Medium
7	Evanoff (2020)	USA/COVID-19	Faculty and clinical staff at medical university	Cross-sectional survey, n = 5500 (60.3)	Current work status/clinical setting/caring for patients with COVID-19/supervisor behaviors supportive of family roles	Stress, anxiety, depression, exhaustion, overall well-being	Being exposed to COVID-19 and having a supervisor who was not supportive of family roles were associated with most of the negative mental health outcomes in the overall and the specific clinical sample. Working as a clinician was associated with more anxiety and decreased overall well-being compared to other occupational groups. Clinical staff working in high-risk (for COVID-19) settings had more negative mental health outcomes than clinical staff that did not work in high-risk settings.	Clinical groups vs. nonclinical groups, high-risk vs. non high-risk clinical groups	Medium
8	Fiksenbaum (2006)	Canada/ SARS	Nurses	Cross-sectional survey, n = 333 (95%)	Perceived SARS threat, organizational support	Emotional exhaustion (MBI-GS)	Working conditions contributed significantly to higher perceived SARS threat, which was associated with increased emotional exhaustion. Higher levels of organizational support were associated with lower perceived SARS threat and emotional exhaustion.	None	Medium
9	Foo (2006)	Singapore/ SARS	Nurses, doctors, assistants at hospital	Cross-sectional survey, n = 322 (86%)	Use of PPE (N95 mask, gloves, gown)	Adverse skin reactions	All those reporting adverse reactions wore N95 masks for a mean 8 h/day mean 8.4 months or gloves for a mean 6.2 h/day mean 9.4 months Staff using other masks and plastic gloves, respectively, did not report adverse skin reactions.	Staff who reported acne, dry skin, and itch were younger than those without reactions	Medium
10	Hacimusalar (2020)	Turkey/ COVID-19	HCWs and non-HCWs	Cross-sectional survey, n = 1121 (HCWs), n = 1035 (non-HCWs) (not reported)	Working hours	Anxiety, hopelessness	More anxiety and hopelessness among HCWs than non-HCWs; more anxiety and hopelessness among nurses than other groups. Association between high working hours and anxiety.	HCWs vs. non-HCWs; nurses vs. doctors	Medium
11	Han (2020)	China/ COVID-19	Nurses in a Chinese province	Cross-sectional survey, n = 21,199 (99%)	Hospitals with cases of COVID-19	Anxiety, depression	Nurses who worked in designated hospitals tended to have higher anxiety scores.	Staff in hospitals with and without COVID-19 patients	High
12	Hoffman (2020)	USA/ COVID-19	Oncology radiation staff	Cross-sectional survey n = 575 (69%)	Working from home	Burnout symptoms	In employees working from home at least part of the time, 74% reported the experience to be positive, and rating the experience as positive was associated with less burnout. Unfavorable work-from-home responses were, in qualitative responses, linked to child/family care and IT issues.	Occupations within oncology radiation	Low
13	Hongling (2020)	China/ COVID-19	Nurses	Cross-sectional survey, n = 159 (66%)	Working at COVID-19 ward	Traumatization, stress	Nurses who worked on the non-critical care ward scored higher on traumatization and stress than nurses who worked on the critical care ward.	Nurses in critical vs. non-critical ward	Low
14	Hoseinabadi (2020)	Iran/ COVID-19	Nurses	Cross-sectional survey, n = 245 (48%)	Working at COVID-19 ward	Burnout	Nurses working at the frontline were more likely to suffer from job stress and burnout than nurses on the usual ward. Job stress was associated with burnout.	Nurses working in frontline vs. usual ward	Medium
15	Huang (2020)	China/ COVID-19	HCWs in radiology departments	Cross-sectional, n= 377 (59%)	Contact with suspected/confirmed COVID-19 patients, availability of PPE, knowledge about COVID-19	Anxiety	A nursing role and lack of PPE were associated with anxiety in multivariate analysis.	Occupations within radiology	High
16	Jiang (2020)	China /COVID-19	Nurses and doctors at hospitals	Cross-sectional survey, n = 4308 (88%)	Use of PPE	Skin injuries	Daily wearing time and grade 3 PPE (N95/KN95 masks, gowns, gloves, and shoes) were associated with skin injuries.	Male gender was associated with skin injuries	Medium
17	Jung (2020)	South Korea/ MERS	Nurses	Cross-sectional survey, n = 147 (100%)	Level of involvement in SARS patients	PTSD	Level of involvement in the care for patients with suspected or confirmed MERS was associated with levels of PTSD.	Several	Medium
18	Khalafallah (2020)	USA/ COVID-19	Neurosurgeons	Cross-section survey, n = 407 (11.3%)	Working in a hostile or difficult environment spending increased time conducting non-neurosurgical medical care due to COVID-19	Burnout	Burnout was associated with working in a hostile or difficult environment (OR = 2.534, *p* = 0.008), and spending increased time conducting non-neurosurgical medical care (OR = 2.362, *p* = 0.019) since the rise of COVID-19.	None	Medium
19	Khalid (2016)	Saudi Arabia/ MERS	HCWs who worked in high-risk areas	Cross-sectional survey, n = 117 (76%)	Safety of self, colleagues, family members	Stress	HCWs who saw their own, their colleagues, and their family members’ safety and well-being threatened experienced stress.	None	Low
20	Khanal (2020)	Nepal/ COVID-19	HCWs	Cross-sectional survey, n = 475 (53%)	Working overtime, insufficient precautionary measures, stigma, work schedule, working in affected district	Anxiety, depression, insomnia	Experienced stigma was associated with all mental health outcomes; inadequate precautionary measures associated with anxiety and depression. Nurses experienced more anxiety than other health professionals.	Nurses/Doctors/Other health professionals	Medium
21	Kim (2016)	Korea/ MERS	ED nurses	Cross-sectional survey, n = 215 (94%)	Job stress, poor hospital resources for treatment of MERS, shift, care for MERS patient	Burnout	ED nurses’ burnout was associated with job stress and poor hospital resources for the treatment of MERS-CoV.	None	High
22	Koksal (2020)	Turkey/ COVID-19	HCWs	Cross-sectional survey, n = 702 (70%)	Workload, COVID-19 training, Contact with COVID-19 patient, unnecessary use of PPE	Symptoms of depression and anxiety	Unnecessary use of PPE was associated with depressive symptoms; increased workload was associated with symptoms of anxiety.	None	Low
23	Kuo (2020)	Taiwan/ COVID-19	Doctors, nurses, medical examinators, administrators	Cross-sectional survey, n = 752 (89%)	Various stressors among HCWs caring for patients with highly infectious disease	Discomfort, burden, etc.	Highest scores were found for the subscales discomfort caused by PPE and burden caring for patients.	Different occupations	Low
24	Lam (2020)	China/ COVID-19	Nurses, physicians, and others from various cities and hospitals	Cross-sectional survey, n = 932 (63–83% in 3 samples)	Infection, PPE	Depression	Most strongly associated with depression were feeling susceptible to contracting COVID-19 and difficulty obtaining face masks.	None	Medium
25	Lan (2020)	China/COVID-19	Physicians and nurses	Cross-sectional survey n = 542 (sex not reported)	Wearing PPE	Skin damage	The prevalence was 97%. Wearing N95 masks or goggles >6 h per day and hand hygiene >10 times per day increased the risk of skin damage.	None	Medium
26	Lancee (2008)	Canada/ SARS	HCWs in Toronto where most SARS patients in Canada were	Cross-sectional retrospective survey and interview, n = 133	Perception of the adequacy of training, protection, and support with respect to SARS	Diagnosed psychiatric disorder	New episodes of psychiatric disorders were directly associated with a history of having a psychiatric disorder before the SARS outbreak and inversely associated with years of healthcare experience and the perceived adequacy of training and support.	None	Medium
27	Lee (2018)	South Korea/ MERS	HCWs	Repeated survey, n = 359 (82%)	MERS-related tasks	Stress and psychological impact	Medical staff that performed MERS-related tasks showed the highest risk for post-traumatic stress disorder symptoms even after time had elapsed.	Different occupations	Medium
28	Liu (2009)	China/ SARS	HCWs	Case-control study, n = 51 cases (69%) and n = 426 controls (69%)	Types of contact with patients; emergency care experience; wearing mask, glasses, protective clothes, etc.; taking training	Diagnosis of SARS according to WHO’s criteria; confirmed with Ig G antibodies against SARS-CoV	Factors significantly associated with increased risk of SARS infection: not wearing a 16-layer or 12-layer cotton surgical mask; emergency care experience; contact with respiratory secretion; not taking training; and contact with chest compression.	None	High
29	Loeb (2004)	Canada/ SARS	Critical care nurses	Case-control study, n = 8 cases (100%) and n = 32 controls (100%)	Types of patient care activities, use of PPE	Suspected or probable SARS case according to Canada’s case definition; confirmed with antibody testing	Activities related to intubation increased SARS risk and use of a mask (particularly an N95 mask) was protective.	None	Medium
30	Luceno-Moreno (2020)	Spain/ COVID-19	HCWs in contact with COVID-19 patients	Cross-sectional survey, n = 1422 (86%)	Type of shift	Post-traumatic stress, anxiety, and depression	Working 12- or 24-h shifts, compared with a large range of other shifts, was associated with mental health outcomes.	Type of healthcare center and occupation	Medium
31	Maraqa (2020)	Palestine/ COVID-19	Frontline HCWs	Cross-sectional survey, n = 430 (55%)	Contact with COVID-19 patients, knowledge, training in outbreak response	Perceived stress level	No training in outbreak response was associated with higher stress levels.	None	Low
32	Marjanovic (2007)	Canada/ SARS	Nurses	Cross-sectional survey, n = 333, (95%)	Organizational support, trust in equipment	Burnout	Higher levels of organizational support and trust in equipment/infection control, and lower levels of contact with SARS patients and time spent in quarantine were associated with lower levels of emotional exhaustion.	None	Medium
33	Matsuo (2020)	Japan/ COVID-19	HCWs	Cross-sectional survey, n = 312 (72%)	Workload, transmission risk	Burnout	Not being a physician, desire for a reduced workload, and desire for appreciation or respect were associated with higher OR for burnout.	Occupation	Medium
34	Maunder (2006)	Canada/ SARS	HCWs in 9 hospitals that treated SARS patients (Toronto) and 5 hospitals that did not (Hamilton)	Cross-sectional survey distributed 1–2 years after the SARS outbreak, n = 769 (86–90%)	Training, PPE, support, conflicts, workload, overtime, stigma	Burnout, Psychological distress, PTSD	Toronto HCWs reported significantly higher levels of burnout, psychological distress, and post-traumatic stress. Toronto workers were more likely to have reduced patient contact and work hours and to report behavioral consequences of stress. Variance in adverse outcomes was explained by a protective effect of the perceived adequacy of training and support and by a provocative effect of a maladaptive coping style and other individual factors.	Hospitals that treated SARS patients vs. hospitals that did not	Medium
35	Mo (2020)	China/ COVID-19	Nurses	Cross-sectional survey, n = 180 (90%)	Working hours per week	Work stress	Higher number of working hours per week was associated with more stress.	None	Medium
36	Monterrosa-Castro (2020)	Colombia/ COVID-19	General practitioners	Cross-sectional survey, n = 531 (60%)	Feeling protected by employer, job satisfaction, stigma, etc.	Generalized anxiety disorder (GAD)	Feeling protected by their employer and job satisfaction were negatively associated with GAD; social discrimination for working as a general practitioner was positively associated with GAD.	None	Medium
37	Morcuende (2020)	US/COVID-19	Physicians	Cross-sectional questionnaire, n = 105 (57%)	Patient work with/without adequate PPE	COVID-19-like symptoms; antibody testing	Exposed and unexposed respondents did not differ regarding COVID-19 antibodies.	None	Medium
38	Morgantini (2020)	60 countries (including Sweden)/ COVID-19	HCWs	Cross-sectional survey, n = 2707 (not reported)	Work impacting household activities, feeling pushed beyond training, exposure to COVID-19 patients, adequate PPE	Burnout	Burnout was associated with work impacting household activities, feeling pushed beyond training, exposure to COVID-19 patients, and making life-prioritizing decisions. Adequate personal protective equipment (PPE) was protective against burnout. Burnout was higher in high-income countries (HICs) compared to low- and middle-income countries (LMICs).	High-income countries (HICs) compared to low- and middle-income countries (LMICs)	Medium
39	Mosheva (2020)	Israel/ COVID-19	Physicians	Cross-sectional survey, n = 1106 (49%)	Pandemic-related stress factors	Anxiety	Lack of knowledge about prevention and protection was associated with anxiety.	None	Medium
40	Nickell (2004)	Canada/ SARS	HCWs	Cross-sectional survey, n = 2001 (79%)	Occupation	Emotional distress	Being a nurse, part-time employment, and the ability to do one’s job affected by the precautionary measures were associated with emotional distress.	Occupation	Medium
41	Ong (2020)	Singapore/ COVID-19	Nurses, doctors, and paramedics	Cross-sectional survey n =158 (70%)	Use of PPE	Headache	PPE usage for >4 h/day was associated with de novo headache.	None	Medium
42	Pratt (2009)	Canada/ SARS	Nurses from several areas of healthcare practice	Cross-sectional survey, n = 536 (97%)	Effort-reward imbalance	Burnout, compliance with infection control	Effort-reward imbalance was associated with burnout and with compliance with infection control measures.	None	Medium
43	Ramaci (2020)	Italy/ COVID-19	HCWs in large hospital in southern Italy	Cross-sectional survey, n = 260 (50%)	Stigma, job demands	Burnout, fatigue	Stigma and job demands were associated with burnout and fatigue.	None	Medium
44	Reynolds (2006)	Vietnam/ SARS	Hospital workers	Cohort study, nested case-control study, n = 153 (n.a.)	Activities during SARS patient’s hospitalization	SARS-CoV infection (confirmed by RT-PCR test or antibodies)	Proximity to index patient was nearly universal among those who were infected. Activities associated with infection risk: touched index patient, came within 1 m, spoke with index patient, saw (viewed) the patient, etc.	None	Medium
45	Rodriguez (2020)	USA/ COVID-19	Academic emergency medicine physicians	Cross-sectional survey, n = 426 (45%)	Several stressors	Stress and burnout	The most commonly cited measures that would alleviate stress or anxiety were increasing personal protective equipment (PPE) availability, offering rapid COVID-19 testing at physician discretion, providing clearer communication about COVID-19 protocol changes, and assuring that physicians can take leave for care of family and self.	None	Low
46	Rossi (2020)	Italy/ COVID-19	HCWs	Cross-sectional survey, n = 1379 (77%)	Several stressors	Post-traumatic stress symptoms (PTSS), insomnia, depression	Being a frontline HCW was associated with PTSS. General practitioners were more likely to endorse PTSS than other HCWs while nurses and healthcare assistants were more likely to endorse severe insomnia. Having a colleague deceased, hospitalized, or in quarantine was associated with negative health outcomes. Being exposed to contagion was associated with symptoms of depression.	Occupation	Medium
47	Ruiz-Fernandez (2020)	Spain/ COVID-19	Nurses and physicians	Cross-sectional survey, n = 506 (77%)	Occupation	Compassion fatigue (CF), burnout (BO), compassion satisfaction (CS), perceived stress (PS)	Physicians had higher CF and BO scores while nurses had higher CS scores.	Occupation	Medium
48	Sampaio (2020)	Portugal/ COVID-19	Nurses	Cross-sectional survey n = 767 (81%)	Overtime work, inadequate PPE	Depression, anxiety, stress	Overtime work and inadequate quantity and quality of PPE were associated with higher levels of depression, anxiety, and stress.	None	Medium
49	Saricam (2020)	Turkey/ COVID-19	Nurses	Cross-sectional survey n = 123 (74%)	Working in COVID-19 ward, regular ward, ICU	Anxiety	COVID-19-related anxiety was associated with working in the wards rather than ICUs.	None	Medium
50	Shah (2020)	UK/ COVID-19	Obstetrics and gynecology doctors	Cross-sectional survey n = 207 (81%)	Working during COVID-19	Depression and anxiety	Obstetricians and gynecologists had more depression and anxiety compared to UK-wide estimates.	Anxiety was more common amongst female doctors compared to males	Medium
51	Shalhub (2020)	58 countries Mainly the US 43% and Brazil 43%/COVID-19	Vascular surgeons	Cross-sectional survey n = 1609	COVID-19 related stressors	Anxiety	Staying separate from family/home and using PPE were associated with increased anxiety. Hospital support was associated with decreased anxiety.	None	Low
52	Singh (2020)	India/ COVID-19	Physicians, nurses, and paramedics	Cross-sectional structured interview by telephone n = 43	Use of PPE	Dermatoses	Descriptive results: Irritant contact dermatitis 39.5%, friction dermatitis 25.5%.	None	Low
53	Smith (2020)	Canada/ COVID-19	HCWs (not specified)	Cross-sectional survey n = 5988 (91%)	PPE and infection control procedures needs met	Anxiety and depression	Higher prevalence of anxiety and depression (using cut-offs) in groups with unmet needs.	None	Medium
54	Styra (2008)	Canada/ SARS	Mainly nurses	Cross-sectional survey n = 248 (86%)	Contact with SARS patients	Post-traumatic stress syndrome	Working in high-risk units was associated with greater distress. HCWs who experienced greater contact with SARS patients while working in high-risk units were less distressed.	Non-SARS units	Medium
55	Su (2007)	Taiwan/ SARS	Nurses	Prospective longitudinal design n = 102	Contact with SARS patients	Psychiatric morbidity and psychological adaptation	Occurrence of psychiatric symptoms was associated with direct exposure to SARS patient care, previous mood disorder history, younger age, and perceived negative feelings	Non-SARS unit nurses	Medium
56	Suryavanshi (2020)	India/ COVID-19	Physicians, nurses, residents, paraclinical	Cross-sectional survey n = 197 (51%)	Knowledge, manpower, fear of infection, pressure, concerns about patient death rates, discrimination	Depression, anxiety	Work environment stressors, such as lack of knowledge, lack of manpower, and fear of infection, were associated with increased risk of combined depression and anxiety.	None	Medium
57	Tabah (2020)	Australia/ COVID-19	Physicians, nurses, and assistants	Cross-sectional survey n = 2711 (46%)	Duration of PPE use, measured as length of shift	Adverse effects (heat, headaches, etc.)	Adverse effects of PPE were associated with longer shifts.	None	Low
58	Tam (2004)	Hongkong/ SARS	Nurses, physicians, assistants	Cross-sectional survey n = 652 (79%)	Contact with SARS patients, employer support	Stress, psychological morbidity	Direct contact with SARS patients was associated with high stress; perceived inadequacy of support items was associated with psychological morbidity.	Nurses had higher stress and more psychological morbidity compared with other professionals	Medium
59	Teleman (2004)	Singapore/ SARS	Doctors, nurses, others	Case-control design with telephone interviews n = 86 (95%)	Contact with SARS patients	SARS infection	Contact with respiratory secretions associated with higher OR. Hand washing and wearing N95 masks associated with lower OR. No effect of wearing gowns or gloves.	None	Medium
60	Wang (2020)	China/ COVID-19	Doctors, nurses, others	Cross-sectional survey n = 1049 (86%)	Contact with COVID-19 patients	Depression anxiety, insomnia	Contact with COVID-19 patients was associated with anxiety and depression, stress, and insomnia.	High-risk vs. low-risk exposure group	High
61	Wang (2020)	China/ COVID-19	Nurses, doctors, others	Cross-sectional survey n = 1234 (90%)	Contact with COVID-19 patients	Stress	Stress was associated with being a nurse, being married, and spending more than 20 days caring for COVID-19 patients. Stress had a negative correlation with being rescue staff.	None	Medium
62	Xiao (2020)	China/ COVID-19	Physicians, nurses	Cross-sectional survey n = 958 (67%)	Access to PPE	Anxiety and depression	Access to PPE was associated with lower levels of anxiety and depression.	Females and those with more contact history had more anxiety and depression	Medium
63	Zerbini (2020)	Germany/ COVID-19	Nurses, physicians	Cross-sectional survey n = 110 (70%)	Contact with COVID-19 patients	Psychosocial burden	Nurses working on the COVID-19 wards reported higher levels of stress, exhaustion, and depressive mood, and lower levels of work-related fulfilment compared to nurses working on regular wards. No difference between groups for physicians.	Nurses vs. physicians	Medium
64	Zhan (2020)	China/ COVID-19	Nurses	Cross-sectional survey n = 2667 (97%)	Working hours	Fatigue	Longer working hours were associated with nurses’ fatigue, and a higher frequency of weekly night shifts had a low positive correlation with nurses’ fatigue.	None	Medium
65	Zhang (2020)	China/ COVID-19	Nurses, doctors, paramedics	Cross-sectional survey n = 1357 (47%)	Overworked before COVID-19	Fatigue	Being overworked before COVID-19 was associated with fatigue after the outbreak.	Frontline vs. non-frontline HCWs	Low
66	Zhang (2020)	Bolivia, Ecuador, Peru/ COVID-19	Nurses, physicians, pharmacists	Cross-sectional survey n = 712 (68%)	Organizational support	Anxiety, life satisfaction	Development and testing of the questionnaire “COVID-19 organizational support”. Identified 3 factors predicting HCWs’ anxiety and life satisfaction: work support, family support, and risk support.	None	Low
67	Zhou (2020)	China/ COVID-19	Firstline hospital staff and general population	Cross-sectional comparative study n = 606 and 1099	Years of working, daily working hours	Depression, anxiety, somatization symptoms, insomnia, suicide risk	More depression, anxiety, somatization, and insomnia in frontline medical staff than in the general population. In frontline medical staff, daily working hours were positively associated with all psychological disorders.	None	Medium

HCWs: Healthcare workers; PPE: Personal protective equipment.

**Table 3:** Overview of quantitative studies assessing interventions to change the work environment or health in the healthcare industry during an epidemic or pandemic (COVID-19, SARS, or MERS, research question 3).

No	Author (Year)	Country	Population	Design, N (% Women)	Comparison Groups	Intervention	Outcome Measure	Effect/Change	Subgroup Comparison	Overall Quality
1	Chen (2006)	Taiwan	116 nursing staff	Before-after design (98)	None	Epidemic prevention plan: in-service training, manpower allocation, PPE, and mental health team	Anxiety, depression, and sleep quality	Anxiety and depression decreased from before to after the intervention, and sleep quality improved.	None	Medium
2	Rogers (2020)	USA	Various HCWs at 10 hospitals	Before-after design 25 Observations 216 Focus groups 72	None	Educational program	Knowledge and practice of respiratory protection	Knowledge increased to typically 100%. Observations showed improper use of respirators (75% of all observations). Focus groups and logged incidents identified competences needed: 1. Know when PPE is needed; 2. Know the policy. 3. Other specific knowledge.	None	Medium
3	Saqib (2020)	UK	HCWs (not specified)	Before-after design n = 93	None	Quiet room at the hospital to recover	Mood	Mood improved after visiting the quiet room.	None	Low
4	Stirling (2015)	Saudi Arabia	HCWs and students	Before-after design n = 75 staff, 65 students	None	Theoretical education about pandemics and precautions	Knowledge	Knowledge improved but still gaps. Note: the results are not reported in numbers!	None	Low
5	Suppan (2020)	Switzerland	Emergency hospital personnel	RCT	Randomized controlled trial	E-learning module on PPE	Knowledge and attitude toward PPE	Correct choice of PPE was significantly increased in both the e-learning and control group, and higher in the e-learning group (but the difference between groups was not significant).	Similar effect regardless of profession or history of COVID-19	High
6	Yen (2006)	Taiwan	Doctors, nurses, admin personnel, and volunteers	Non-randomized study with comparison group n = 459	Other Taiwan hospitals	Triage, risk zones, alcohol dispensers	Infected with SARS	Less infected staff (0.03 cases/bed) compared with staff at comparison hospitals (0.13 cases/bed).	None	Medium

HCWs: Healthcare workers; PPE: Personal protective equipment.

**Table 4:** Overview of qualitative and mixed-methods studies exploring how the work environment in the healthcare sector is affected by an epidemic or pandemic (research question 1) and studies investigating the associations between the work environment and health during an epidemic or pandemic (research question 2).

No	Author (Year)	Country	Epidemic/Pandemic	Sample	Phenomenon Explored	Data Collection and Analytic Method	Main Results	Overall Quality
1	Algunmeeyn (2020)	Jordania	COVID-19	10 nurses, 10 physicians, 10 pharmacists	Factors influencing healthcare providers’ burnout	Qualitative study Individual interviews Thematic analysis	Three themes: 1. Job stress; 2. Staff and resource adequacy; 3. Fear of COVID-19 infection.	Low
2	Bergeron (2006)	Canada	SARS	941 community nurses	Influence on work and personal lives	Mixed methods study Questionnaire. Thematic analysis with some quantitative descriptives	Two themes: 1. Experience: 66% of respondents cited increased hours and weekend shifts, increased paperwork, staff shortages, program stoppages, and additional work relating to patient and visitor screening and the mandatory use of gowns and masks. 2. Learning from the experience: opportunities for personal learning, professional and policy development, and insight into policy and administrative implications.	Medium
3	De Wit (2020)	Canada	COVID-19	468 emergency physicians and residents	Burnout time trends (quantitative) Sources of psychological stress (qualitative)	Mixed-methods study Weekly online survey including open-ended questions Hierarchical logistic regressions Thematic analysis	No time trend in burnout levels (10–18% over 10 weeks). Number of shifts per week and tested for COVID-19 (positive or negative) were positively associated with burnout. Two themes: 1. Impact of COVID-19 on the work environment: personal safety, academic and educational work, PPE, workforce, patient volumes, work patterns, work environment; 2. Fears about the ramifications of COVID-19 on lifestyle: a new financial reality, contrasting negative and positive experiences.	Medium
4	Gao (2020)	China	COVID-19	14 nurses	Experiences of shift patterns	Qualitative study Thematic analysis	Four themes: 1. Assess the competency of nurses to assign nursing work scientifically and reasonably; 2. Reorganize nursing workflow to optimize shift patterns; 3. Communicate between managers and frontline nurses to humanize shift patterns; 4. Nurses’ various feelings and views on shift patterns.	Medium
5	Kackin (2020)	Turkey	COVID-19	10 nurses	Psychosocial problems	Qualitative study Individual interviews Thematic analysis	Three themes: 1. Effects of the outbreak: Working conditions, psychological, social; 2. Short-term coping strategies; 3. Needs. Working conditions concerned: lack of equipment, unfairness in work distribution, change in the working unit, process management, being appreciated as healthcare personnel, difficulty in working with different team members, decreased quality of care, obligation to make ethical decisions, and the risk of infection due to frequent contact in nursing.	Medium
6	Kang (2018)	South Korea	MERS	27 nurses	Working experiences	Qualitative study Focus groups and individual interviews Content analysis	Four themes: 1. Experiencing burnout due to the heavy workload; 2. Relying on personal protective equipment for safety; 3. Being busy with catching up with the new guidelines related to Middle East respiratory syndrome; 4. Caring for suspected or infected patients with caution.	Medium
7	Karimil (2020)	Iran	COVID-19	12 nurses	Caring for patients	Qualitative study Individual interviews Thematic analysis	Three themes: 1. Mental condition (subthemes anxiety/stress and fear); 2. Emotional condition (subthemes suffering/affliction and waiting for death); 3. Care context (subthemes turmoil and lack of support/equipment). Work pressure, inexperience, chaos, and staff shortage.	Medium
8	Lee (2020)	South Korea	MERS	17 nurses	Caring for patients	Qualitative study Individual interviews Thematic analysis	Themes: 1. Fear of uncertainty (infection, novel equipment); 2. Beyond hesitation; 3. A scene like a battlefield (difficulties because of PPE); 4. Chaotic nursing identity; 5. Buttresses for sustainability; 6. Lingering trauma; 7. Expanded horizon of nursing.	Medium
9	Liu (2020)	China	COVID-19	9 nurses, 4 physicians	Combating COVID-19	Qualitative study Individual interviews Thematic analysis	Three themes: 1. Being fully responsible for patients’ wellbeing—’this is my duty’. Healthcare providers volunteered and tried their best to provide care for patients. Nurses had a crucial role in providing intensive care and assisting with the activities of daily living. 2. Challenges of working on COVID-19 wards. Healthcare providers were challenged by working in a totally new context, exhaustion due to heavy workloads and protective gear, the fear of becoming infected and infecting others, feeling powerless to handle patients’ conditions, and managing relationships in this stressful situation. 3. Resilience amid challenges. Healthcare providers identified many sources of social support and used self-management strategies to cope with the situation. They also achieved transcendence from this unique experience.	Medium
10	Liu (2020)	China	COVID-19	17 nurses	Combating COVID-19	Qualitative study Individual interviews Thematic analysis	Four themes: 1. Facing tremendous new challenges and danger; 2. Strong pressure because of fear of infection, exhaustion by heavy workloads, and stress of nursing seriously ill COVID-19 patients; 3. Strong sense of duty and identity as a healthcare provider; 4. Rational understanding of the epidemic—the nurses believed that the epidemic would soon be overcome and would like to receive disaster rescue training.	Medium
11	Mahendran (2020)	Hong Kong	COVID-19	120 staff at dental teaching hospital	Health outcome Generalized Anxiety Disorder (quantitative) Psychosocial implications of COVID-19 (qualitative)	Mixed-methods study Survey with closed and open questions Descriptive statistics Thematic analysis	Severe GAD in 16.7%. No access to PPE for 33%. The most prevalent concerns: Friends and family (24%); Personal health 11%; Nature of disease 11%; Current job 10%; General uncertainty 9%; Social and mental health 9%; Personal protection 8%.	Medium
12	McBeath (2020)	UK	COVID-19	335 psychotherapists	Experiences and challenges of working remotely	Mixed-methods study Survey with closed and open questions Descriptive statistics Thematic analysis	Remote work was perceived as challenging by 80% but reported to be the future core business by 65% Three themes: 1. Adaption issues: less job satisfaction, difficult technology, developed strategies to pace work; 2. Opportunities: less travel, less family–work conflict; 3. Challenges: more fatigue and strain, uncertainty about clinical effectiveness.	High
13	Mohindra (2020)	India	COVID-19	574 HCWs at a tertiary care hospital	Experience of social and emotional distancing	Mixed-methods study Survey with closed- and open-ended questions Descriptive statistics	Four predefined affected domains: 1. Hospital: avoided by colleagues (51%); 2. Neighborhood: avoided and verbally assaulted (54%); 3. Family and home: avoided and verbally assaulted (34%); 4. Self: anxious and guilty (99%).	Medium
14	O’Connor (2009)	Canada	SARS	100 nurses	Identify gaps in risk communication	Qualitative study Focus groups	Key areas in which risk communication could be more efficient to address nurses’ concerns: 1. Managing uncertainty; 2. Occupational health and safety; 3. Employee quality of life. High levels of uncertainty, lack of trust, and questions about leadership credibility emerged as important risk communication challenges. Communication problems were compounded by a lack of reliable information, frequent changes in infection control guidelines and risk avoidance messages, and contradictory actions of management and senior leaders.	Medium
15	O’Sullivan (2009)	Canada	SARS	100 nurses	Need for organizational and social support	Qualitative study Focus groups	Four themes: 1. Personal/professional dilemmas; 2. Assistance with child, elder, and/or pet care; 3. Adequate resources and vaccinations to protect families; 4. Appropriate mechanisms to enable two-way communication between employees and their families under conditions of quarantine or long work hours.	Medium
16	Robertson (2004)	Canada	SARS	10 hospital healthcare workers of mixed professions	Psychosocial effects of being quarantined	Qualitative study Individual interviews Grounded theory	Three themes: 1. Loss: restricted physical contact, wearing a mask, remaining at home; 2. Duty: but anxiety when caring for infected patients; 3. Conflicts: fear of infecting the family. Quarantined workers experienced stigma, fear, and frustration. We highlight the need for clear and easily accessible information on dealing with infectious diseases.	Medium
17	Sadati (2020)	Iran	COVID-19	24 nurses	Experiences of the COVID-19 outbreak	Qualitative study Individual interviews Content analysis	Five themes: 1. Defected preparedness (lack of PPE); 2. The worst perceived risk (infection risk); 3. Family protection; 4. Social stigma (avoided by family and others); 5. Sacrificial commitment (committed to their work).	Medium
18	Sethi (2020)	Pakistan	COVID-19	290 healthcare workers in the private and public sector, including medicine and medicine education and dentistry	Personal and professional impact	Qualitative study Open-ended questions in questionnaire Thematic analysis	1. Personal impact; 2. Professional impact: increased workload, financial instability; 3. Challenges: managing home and family, lack of PPE.	Low
19	Sun (2020)	China	COVID-19	20 nurses	Psychological experiences	Qualitative study Individual interviews Thematic analysis	Four themes: 1. Negative emotions (fatigue, discomfort, and helplessness) were caused by high-intensity work, fear and anxiety, and concern for patients and family members. 2. Self-coping styles: psychological and life adjustment, altruistic acts, team support, and rational cognition. 3. Growth under pressure: increased affection and gratefulness, development of professional responsibility and self-reflection. 4. Positive and negative emotions occurred simultaneously.	Medium
20	Xu (2020)	China	COVID-19	21 primary care practitioners	Barriers to and experiences of COVID-19 epidemic control	Qualitative study. Individual telephone interviews. Thematic analysis.	Challenges: 1. Inappropriate scheduling and role ambiguity; 2. Difficult tasks and inadequate capacities; 3. Unexperienced community workers and insufficient cooperation. The practitioners perceived respect and a sense of accomplishment and were preoccupied with the outbreak. Others were frustrated by fatigue and psychological distress. Suggestions were made for improving management, optimizing workflows, providing additional support, facilitating cooperation, and strengthening the primary care system.	Medium
21	Zhang (2020)	China	COVID-19	23 nurses	Nurses’ change process during the care of patients with COVID-19	Qualitative study Individual interviews Thematic analysis	1. Early stage (from notice to entering the isolation unit): Ambivalence. Torn between professional mission and fear of being infected. 2. Middle stage (after 1–2 weeks at unit): Emotional exhaustion due to the unfamiliar working environment and colleagues, wearing PPE, isolated loneliness, fear of getting infected. 3. Later stage (after 3–4 weeks at unit): Energy renewal due to adaptation to the new working environment, mutual support from team, social support, monetary incentives, and recognition from the government and public.	Low

HCWs: Healthcare workers; PPE: Personal protective equipment.

**Table 5:** Overview of quantitative studies assessing how the work environment in organizations outside the healthcare sector is affected by an epidemic or pandemic (research question 1). All studies are in the context of COVID-19.

No	Author (Year)	Country	Population	Design, n (% Women)	Comparison Groups	The Effect on Work Environment Measure	Subgroup Comparisons	Overall Quality
1	Craig (2020)	Australia	Dual-earner parent couples	Cross-sectional survey, n = 1536 (n.a.)	Retrospectively self-reported pre-COVID-19 and during COVID-19 (about self and partner)	Less than 10% lost their job, were stood down, or found work elsewhere. Most respondents were working at home during pandemics. Earnings decreased. Work hours decreased in paid work and increased in unpaid work. For most respondents, subjective time pressure lessened. A higher proportion were extremely dissatisfied regarding how they divided their time between paid and unpaid work compared to before pandemics. A higher proportion of women were extremely unsatisfied with how they and their partner shared paid and unpaid work compared to before pandemics.	Gender differences narrowed in full-/part-time work, time pressure	Low
2	Priolo Filho (2020)	Brazil	Child protection professionals	Cross-sectional survey, n = 309 (89%)	Self-reported change	Average hours worked per week decreased compared to before pandemics.	None	Medium
3	Yildirim (2020)	France, Germany, Italy, Norway, Sweden, Turkey, UK, US	Academics	Cross-sectional survey, n = 198 (65%)	Men/women	Time spent on work and routines in childcare changed more for women than for men; no difference between men and women in changed routines in housework and change in their contribution to housework.	Having children or not: daily routines of women academics with children were disproportionately affected	Low

**Table 6:** Overview of quantitative studies assessing the associations between work environment factors and health in organizations outside the healthcare sector during an epidemic or pandemic (research question 2). All studies are in the context of COVID-19.

No	Author (Year)	Country	Population	Design, n (% Women)	Exposure	Outcome	The Association between Work Environment and Health	Subgroup Comparisons	Overall Quality
1	Molino (2020)	Italy	Several industries	Cross-sectional survey, n = 743 (59%)	Three dimensions of technostress: overload (e.g., work fast), invasion (e.g., less time with family), complexity (e.g., do not understand the technology)	Behavioral stress	All three dimensions of technostress showed a positive relationship with behavioral stress.	None	Low
2	Moretti (2020)	Italy	Remote-working office staff	Cross-sectional survey, n = 51 (57%)	Working from home	Stress, neck pain, low back pain	Working from home was associated with being less stressed in 39% and more stressed in 33% of the participants; worsening of previous neck pain was reported by 50% and improvement by 8%; worsening of low back pain was reported by 38% and improvement by 14%.	None	Low
3	Sadiq (2020)	Pakistan	Police constables	Cross-sectional survey, n = 247 (0%)	Workload, work–family conflict	Job stress	Workload and work–family conflict were positively associated with job stress.	None	Medium
4	Sasaki (2020)	Japan	Full-time workers	Cross-sectional survey, n = 1379 (49%)	Number of preventive workplace measures taken in response to COVID-19	Fear and worry about COVID-19, psychological distress	The number of preventive workplace measures was positively associated with fear and worry about COVID-19, and negatively associated with psychological distress.	None	Medium
5	Song (2020)	China	Working populations	Cross-sectional survey n = 709 (74%)	Location of work	Anxiety, depression, insomnia	Location of work was not associated with anxiety, depression, and insomnia. Working at home and office alternatively vs. at the office was negatively associated with somatization.	None	Medium
6	Tan (2020)	China	Work force	Cross-sectional survey n = 673 (25%)	Ventilation in the workplace, workplace hygiene, perception that the company cares about your health	Anxiety, depression, insomnia, stress	Having good ventilation at the workplace was not associated with mental health status. Improved workplace hygiene after the COVID-19 outbreak was not associated with anxiety, depression, and insomnia, and was negatively associated with stress. Perception that the company cares about your health was not associated with mental health status.	None	Medium
7	Wong (2020)	Hong-Kong	Full- or part-time-employed or self-employed employees	Cross-sectional survey n = 1048 (68%)			Dissatisfaction with workplace infection control policy and measure was associated with lower self-reported health-related quality of life; the association was mediated by perception of infection risk.	None	Medium

**Table 7:** Overview of qualitative and mixed-methods studies exploring how the work environment outside the healthcare sector is affected by an epidemic or pandemic (research question 1) and studies investigating the associations between the work environment and health during an epidemic or pandemic (research question 2).

No	Author (Year)	Country	Epidemic/ Pandemic	Sample	Phenomenon Explored	Data Collection and Analytic Method	Main Results	Overall Quality
1	Deguchi (2020)	Japan	COVID-19	6 sanitation workers	Impact on daily lives	Telephone interviews Thematic analysis	Seven themes: 1. Alerting overseas news of potential dangers; 2. Fear of contracting COVID-19; 3. Negotiated for safer protocols and gear; 4. Increased workload; 5. Experience of discrimination and stigma; 6. Increased public attention and awareness; 7. Our work goes beyond garbage collection.	Low
2	Gearing (2007)	Canada	SARS	19 social workers (out of 48) at the hospital	Experiences and work practices	Focus groups Thematic analysis	Three themes: 1. Emotional level (emotional awareness and coping strategies); 2. Technical level (communication and advocacy/bridging); 3. Unintended consequences (interrupted education, unsafe at hospital, perception/worries from others).	High
3	Kim (2020)	UK	COVID-19	24 teachers from English state schools	Experiences of partial school closures and lockdown	Individual interviews Thematic analysis	Six themes: 1. Uncertainty (negative emotions, rush, and panic); 2. Finding a way (adjusted thinking and behavior to provide remote teaching); 3. Worry for the vulnerable (pupils with violent homes); 4. Importance of relationships (pupils, parents); 5. Teacher identity (need to organize and plan, meet pupils); 6. Reflections (less busy, flexibility, difficult with home–work balance).	Medium
4	Neary (2020)	USA	COVID-19	67 teachers at Physician Assistant Education	Experiences of adaption to new instructional techniques	Survey with closed and open questions T-test, ANOVA. Thematic analysis	Prior experience with technology was associated with lower levels of stress. Concern about technology was the most common stressor and cause of decreased quality of instruction. Four themes: 1. Support; 2. Time; 3. Logistics; 4. Interaction.	Medium
5	Pather (2020)	Australia	COVID-19	18 university teachers	Disruptions and changes in anatomy education	Individual interviews Thematic analysis	1. Continuing education (loss of integrated “hands-on” experiences); 2. Challenges (workload, traditional roles, students, pedagogy, personal educational philosophies); 3. Key opportunities (enabling synchronous teaching across remote sites, expanding offerings into the remote learning space, and embracing new pedagogies); 4. Managing anatomy education’s transition six critical elements (community care, clear communications, clarified expectations, constructive alignment, community of practice, ability to compromise, adapt, continuity planning).	Medium

## Data Availability

Not applicable.

## Appendix A. Search Strategy

**Table A1:** Database: Cinahl (via Ebsco) Date: 5 October 2020.

Search nr	Search Term	Results
**Population: working population**		
#1	Employees [MH+]	678
#2	Industry [MH+]	49,859
#3	Health Occupations [MH+]	755,520
#4	Occupations and Professions [MH+]	97,601
#5	Women, Working [MH+]	4309
#6	Work [MH+]	7345
#7	Company OR Employ * OR Industry OR Job OR “Occupational group *” OR Occupations OR Personnel OR Staff OR Work * [Title/Abstract]	710,991
#8	#1 OR #2 OR #3 OR #4 OR #5 OR #6 OR #7	1,647,557
**Intervention: Occupational health in the context of coronavirus**		
#9	Coronavirus [MH+]	10,073
#10	Coronavirus Infections [MH+]	17,917
#11	COVID-19 [MH+]	8752
#12	Middle East Respiratory Syndrome Coronavirus [MH+]	425
#13	SARS Virus [MH+]	312
#14	Severe Acute Respiratory Syndrome [MH+]	2293
#15	Corona OR Coronavirus OR cov2 OR COVID19 OR COVID-19 OR MERS OR SARS OR 2019-nCoV * OR “2019-nCoV infection *” [Title/Abstract]	22,197
#16	#9 OR #10 OR #11 OR #12 OR #13 OR #14 OR #15	26,523
#17	Occupational Diseases [MH+]	39,966
#18	Occupational Health [MH+]	62,588
#19	Occupational Health Nursing [MH+]	4552
#20	Occupational Health Services [MH+]	7659
#21	Occupational Medicine [MH+]	459
#22	Occupational Safety [MH+]	20,833
#23	Psychosocial Factors [MH+]	722
#24	Psychosocial Aspects of Illness [MH+]	197,113
#25	Work Environment [MH+]	33,011
#26	Workload [MH+]	15,717
#27	Work Related Illnesses [MH+]	476
#28	Occupational Disease * OR Occupational Health OR Occupational Medicine OR Psychosocial OR Psycho-social OR Stressor * OR (Work OR Working) N3 (Condition * OR Environment * OR Related OR Load) OR Workload [Title/Abstract]	125,105
#29	17 OR 18 OR 19 OR 20 OR 21 OR 22 OR 23 OR 24 OR 25 OR 26 OR 27 OR 28	883,355
#30	#8 AND #16 AND #29	2451

[MH+] = Subject heading, exploded. [Title/Abstract] = Title or abstract. * = Truncation.

**Table A2:** Database: PsycInfo (*via* Ebsco) *Date: 20201005*.

Search nr	Search Term	Results
**Population: working population**		
#1	Occupations [DE]	12,145
#2	Personnel [DE]	10,757
#3	Work Teams [DE]	5151
#4	Working Women [DE]	6477
#5	Company OR Employ * OR Industry OR Job OR “Occupational group *” OR Occupations OR Personnel OR Staff OR Work * [Title/Abstract]	1,042,507
#6	#1 OR #2 OR #3 OR #4 OR #5	1,290,435
**Intervention: Occupational health in the context of coronavirus**		
#7	Coronavirus [DE]	1006
#8	Middle East Respiratory Syndrome [DE]	24
#9	Severe Acute Respiratory Syndrome [DE]	260
#10	corona OR coronavirus OR cov2 OR COVID19 OR COVID-19 OR MERS OR SARS OR 2019-nCoV * OR “2019-nCoV infection *” [Title/Abstract]	3503
#11	#7 OR #8 OR #9 OR #10	4164
#12	Occupational Health [DE]	5907
#13	Occupational Safety [DE]	3649
#14	Psychosocial Factors [DE]	34,687
#15	Psychosocial Outcomes [DE]	233
#16	Working Conditions [DE]	23,362
#17	Work Related Illnesses [DE]	1070
#18	Occupational disease * OR Occupational Health OR Occupational Medicine OR Psychosocial OR Psycho-social OR Stressor * OR (Work OR Working) N3 (Condition * OR Environment * OR Related OR Load) OR Workload [Title/Abstract]	172,647
#19	12 OR 13 OR 14 OR 15 OR 16 OR 17 OR 18	350,431
#20	#6 AND #11 #AND #19	235

[DE] = Thesaurus of Psychological Index Term. [Title/Abstract] = Title or abstract. * = Truncation.

**Table A3:** Database: Pubmed (via NCBI) Date: 2020105.

Search nr	Search Term	Results
**Population: working population**		
#1	Employment [MeSH Terms]	87,290
#2	Industry [MeSH Terms]	316,795
#3	Occupational Groups [MeSH Terms]	610,845
#4	Occupations [MeSH Terms]	34,444
#5	Women, working [MeSH Terms]	5375
#6	Work [MeSH Terms]	63,870
#7	Workplace [MeSH Terms]	23,230
#8	Company OR Employ * OR Industry OR Job OR “Occupational group *” OR Occupations OR Personnel OR Staff OR Work * [Title/Abstract]	2,340,428
#9	#1 OR #2 OR #3 OR #4 OR #5 OR #6 OR #7 OR #8	3,057,511
**Intervention: Occupational health in the context of coronavirus**		
#10	Coronavirus Infections [MeSH Terms]	38,313
#11	SARS Virus [MeSH Terms]	3555
#12	Corona OR Coronavirus OR cov2 OR COVID19 OR COVID-19 OR MERS OR SARS OR 2019-nCoV * OR “2019-nCoV infection *” [Title/Abstract]	83,910
#13	#10 OR #11 OR #12	87,768
#14	Occupational Diseases [MeSH Terms]	132,343
#15	Occupational Health [MeSH Terms]	33,662
#16	Occupational Health Services [MeSH Terms]	10,554
#17	Occupational Medicine [MeSH Terms]	23,330
#18	Occupational Health Nursing [MeSH Terms]	4390
#19	Workload [MeSH Terms]	21,538
#20	Occupational disease * OR Occupational Health OR Occupational Medicine OR Psychosocial OR Psycho-social OR Stressor * OR Work Condition * OR Working Condition * OR Work Environment * OR Working Environment * OR Workload OR Work load OR Work related [Title/Abstract]	232,828
#21	#14 OR #15 OR #16 OR #17 OR #18 OR #19 OR #20	395,316
#22	#9 AND #13 AND #21	1043

[MeSH] = Term from the Medline controlled vocabulary, including terms found below this term in the MeSH hierarchy. Title/Abstract] = Title or abstract. * = Truncation.

**Table A4:** Database: Web of Science Date: 2020105.

**Population: working population**		
#1	Company OR Employ * OR Industry OR Job OR “Occupational group *” OR Occupations OR Personnel OR Staff OR Work * [Topic]	6,936,326
**Intervention: Occupational health in the context of coronavirus**		
#2	Corona OR Coronavirus OR cov2 OR COVID19 OR COVID-19 OR MERS OR SARS OR 2019-nCoV * OR “2019-nCoV infection *” [Topic]	133,497
#3	Occupational Disease * OR Occupational Health OR Occupational Medicine OR Psychosocial OR Psycho-social OR Stressor * OR (“Work” or “Working”) NEAR/3 (Condition * OR Environment * OR Related OR Load) OR Workload [Topic]	459,304
#4	#1 AND #2 AND #3	844

[Topic] = Title, Abstract, Author Keywords, Keywords Plus. * = Truncation.

Quality assessment

Mixed Methods Appraisal Tool (MMAT) 2018, [14], an instrument developed for the quality assessment in systematic reviews including original articles with different research designs, was used. The criteria are listed below.

For quantitative non-randomized studies:

(1) If the participants are representative of the target population, (2) if the measurements for outcome and exposure are appropriate, (3) if outcome data is complete (referring to dropout), (4) if relevant confounders are accounted for, and (5) during the study period, if intervention/exposure occurred as intended.

For quantitative randomized controlled trials:

(1) If randomization was appropriately performed, (2) if the groups are comparable at baseline, (3) if there is complete outcome data, (4) if outcome assessors are blinded to the intervention provided, and (5) if the participants adhered to the assigned intervention.

For qualitative studies:

(1) If the qualitative approach is appropriate to answer the research questions, (2) if the data collection methods are adequate to address the research question, (3) if the findings are adequately derived from the data, (4) if the interpretation of the results is sufficiently substantiated by the data, and (5) if there is coherence between qualitative data sources, collection, analysis, and interpretation

For mixed-methods studies:

(1) If there is an adequate rationale for using a mixed-methods design to address the research questions, (2) if the different components of the study are effectively integrated to answer the research question, (3) if the outputs of the integration of qualitative and quantitative components are adequately interpreted, (4) if divergences and inconsistencies between quantitative and qualitative results are adequately addressed, and (5) if the different components of the study adhere to the quality criteria of each tradition of the methods involved.

We adjusted the quality assessment criteria for quantitative non-randomized studies. Because the last criterium (during the study period, if intervention/exposure occurred as intended) was not relevant for cross-sectional studies, which is the majority of the included studies, we did not consider this criterion in our quality assessment. Thus, only four criteria were used for quantitative non-randomized studies.

The core research team developed a procedure to rate the overall quality of each study as low, medium, or high. The aim was both to identify studies of low quality to exclude these from our analysis and conclusions, and to obtain an overall picture of the quality of the included research. In this procedure, we considered each study design separately and came to the following criteria for the overall study quality.

For quantitative non-randomized studies:

High quality = 4 yes, Medium quality = 2–3 yes, Low quality = 1 yes.

For quantitative randomized controlled trials:

High quality = 5 yes, Medium quality = 3–4 yes, Low quality = 1–2 yes.

For qualitative and mixed methods studies:

High quality = 5 yes, Medium quality = 3–4 yes, Low quality = 1–2 yes.
